# Structures, properties, and functions of the stings of honey bees and paper wasps: a comparative study

**DOI:** 10.1242/bio.012195

**Published:** 2015-05-22

**Authors:** Zi-Long Zhao, Hong-Ping Zhao, Guo-Jun Ma, Cheng-Wei Wu, Kai Yang, Xi-Qiao Feng

**Affiliations:** 1 AML, Department of Engineering Mechanics, Tsinghua University, Beijing 100084, China; 2 Center for Nano and Micro Mechanics, Tsinghua University, Beijing 100084, China; 3 State Key Lab of Structural Analysis for Industrial Equipment, Faculty of Vehicle Engineering and Mechanics, Dalian University of Technology, Dalian, Liaoning 116024, China

**Keywords:** Sting, Honey bee, Paper wasp, Penetration, Mechanical property, Biomimetics

## Abstract

Through natural selection, many animal organs with similar functions have evolved different macroscopic morphologies and microscopic structures. Here, we comparatively investigate the structures, properties and functions of honey bee stings and paper wasp stings. Their elegant structures were systematically observed. To examine their behaviors of penetrating into different materials, we performed penetration–extraction tests and slow motion analyses of their insertion process. In comparison, the barbed stings of honey bees are relatively difficult to be withdrawn from fibrous tissues (e.g. skin), while the removal of paper wasp stings is easier due to their different structures and insertion skills. The similarities and differences of the two kinds of stings are summarized on the basis of the experiments and observations.

## INTRODUCTION

Many animals and plants are equipped with sharp weapons. A few examples are bovine horn (Li et al., 2010, 2011), scorpion stings (Dehghani and Fathi, 2012), caterpillar spines (Ma et al., 2011), mosquito proboscis (Kong and Wu, 2009), and the thorns of genera *Agave*, *Aloe*, and *Euphorbia* (Lev-Yadun, 2003). These sting-like organs have various crucial and intriguing functions. For instance, most spinescence of plants, including spines, thorns, and prickles, can act as a defense against herbivores or to reduce plant digestibility (Hanley et al., 2007). The cactus *Opuntia microdasys*, well-known for its excellent drought tolerance, uses conical spines to collect fog in arid environments (Ju et al., 2012). Despite of their similar functions, these organs have evolved, through natural selection, different macroscopic morphologies and microscopic structures. For example, North American porcupine quills are featured by backward-facing deployable barbs, while African porcupine quills and hedgehog spines are smooth (Vincent and Owers, 1986; Cho et al., 2012).

Understanding the insertion mechanisms of a needle or sting into biological soft tissues is an issue of particular interest in medical engineering (e.g. therapeutic drug delivery, and removal of tissue sample from the body). The penetration behavior of stings and needles have been investigated by theoretical models (Shergold and Fleck, 2004; Misra et al., 2010), experimental measurements (Duan and Messing, 2000; Heverly et al., 2005; Das and Ghatak, 2011), empirical predictions (Davis et al., 2004; Mahvash and Dupont, 2010), and numerical simulations (Brett et al., 1997; DiMaio and Salcudean, 2002; Oldfield et al., 2013). A penetration process can be decomposed into the following three phases (Shergold and Fleck, 2005). Firstly, the advancing sting tip presses and deforms the tissue surface until the pressing force reaches the critical load of tissue puncture. Secondly, the tissue capsule ruptures and the needle continuously inserts the tissue in the steady state. Finally, the sting motion is stopped and the tissue relaxes due to its viscoelasticity. If the sting has a bevel tip, the tissue will be ruptured via a planar mode-I crack and subsequently wedged open by the advancing sting shaft (Azar and Hayward, 2008). The penetration force of the sting can be considered as a superposition of three components, corresponding to tissue stiffness, interfacial friction, and cutting force needed to slice through the tissue, respectively (Okamura et al., 2004).

In contrast to artificial needles, such insect organs as bee stings are naturally endowed with elegant structures and superior mechanical properties, which contribute to their multiple biological functions. For instance, a very low force (∼18 μN) is needed by a mosquito to pierce its proboscis into human skin, which is at least three orders of magnitude smaller than the force for an artificial microneedle with an ultra-sharp tip (Kong and Wu, 2009). Therefore, much effort has been directed towards exploring the relation between the structure and mechanical property of insect stings (Duan and Messing, 2000; Barham, 2007; Aoyagi et al., 2008; Izumi et al., 2011). For example, biomimetic stings have been fabricated for painless transdermal injection (Aoyagi et al., 2008) and drilling technology (Gao et al., 2007).

Bees are thought to have evolved from a wasp ancestor (Winston, 1991), but they have diverged from the latter in many characteristics (Michener, 1974; Giannoni-Guzmán et al., 2014). The stings (highly modified ovipositors) of bees and wasps enable diverse biological and mechanical functions, e.g. attack/predation, defense, location, envenomation, prey carriage, mating, and cutting (Radović, 1985; Radović and Sušić, 1997; Quicke et al., 1999; Kong and Wu, 2010; Frasson et al., 2010; Izumi et al., 2011). The anatomy, histology, phylogeny, and biology of the stings of bees (Pearson, 1900; Snodgrass, 1956; Dade, 1962; Visscher et al., 1996; Packer, 2003; Cardinal and Packer, 2007) and wasps (Akre et al., 1981; Vilhelmsen et al., 2001; Matushkina, 2011; Fortunato and Turillazzi, 2012) have attracted considerable attention. For example, Wu et al. (2014) experimentally demonstrated that barbs facilitate the helical penetration of honey bee stings. It is of significance to investigate the mechanical mechanisms of the stings for the structural optimization and design of biomimetic needles. However, the different structures and properties between the stings of honey bees and paper wasps remain elusive.

In this paper, a comparative study is performed for the stings of honey bees and paper wasps. Their chemical constitutes, structures, and properties were experimentally investigated, and their refined insertion skills were also compared. It is found that the stings of honey bees and paper wasps, though with similar constituents and biological functions, have distinctly different structures and insertion skills.

## RESULTS

**Fig. 1.**
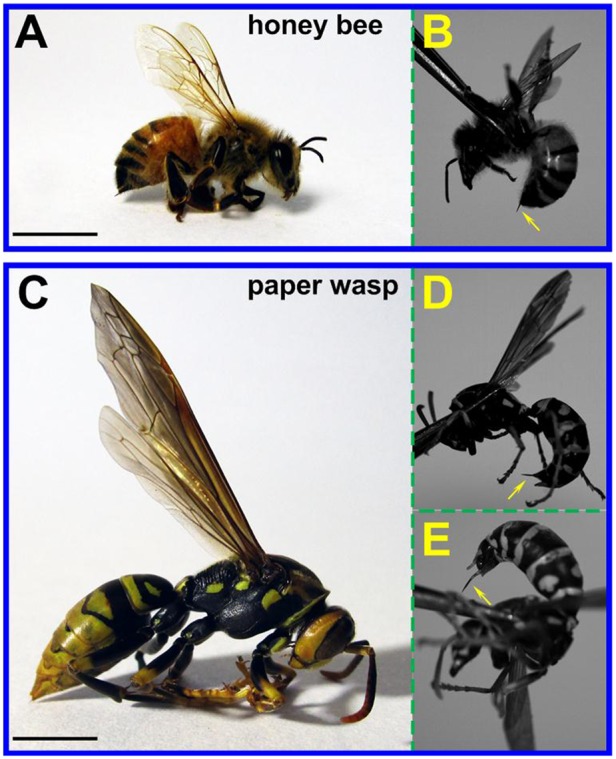
**Honey bee and paper wasp morphology.** (A) A honey bee, (C) a paper wasp, and (B,D,E) the stings of the two species at the maximum thrust, which are indicated by the yellow arrows. Scale bars=5 mm.

The bodies of honey bees and paper wasps have a similar waist-like appearance but they differ in shapes and sizes, as shown in Fig. 1. For instance, a bee has hairy abdomens and flat legs (Fig. 1A), while a wasp has sleek abdomens and round legs (Fig. 1C). The abdomen ventral of a honey bee always keeps flat during penetration, while a paper wasp can swiftly bend the abdomen into a highly curved morphology when it attacks. As a thin junction between their thorax and abdomen, the waist allows flexible movements of the abdomen with respect to the thorax. The bodies of the honey bees are measured to be 90.5±29.5 mg in weight and 11.7±1.3 mm in length, while the paper wasps are 105.4±20.6 mg in weight and 14.7±1.7 mm in length.

The stings of honey bees and paper wasps are commonly held inside a chamber at the rear end of their abdomens. A honey bee can only defense and stab the intruders at its ventral side (Fig. 1B, supplementary material Movie S1), while a paper wasp can attack the enemies at both the ventral (Fig. 1D, supplementary material Movie S2) and dorsal (Fig. 1E, supplementary material Movie S3) sides by flexibly spinning and bending its abdomen. Therefore, it is dangerous to hold the wings of a paper wasp by fingers. The different shapes and flexibilities of the abdomens of the two species might affect their striking scopes, and the morphologies of the stings are also adaptive to their different attacking features.

### Constituents

**Fig. 2.**
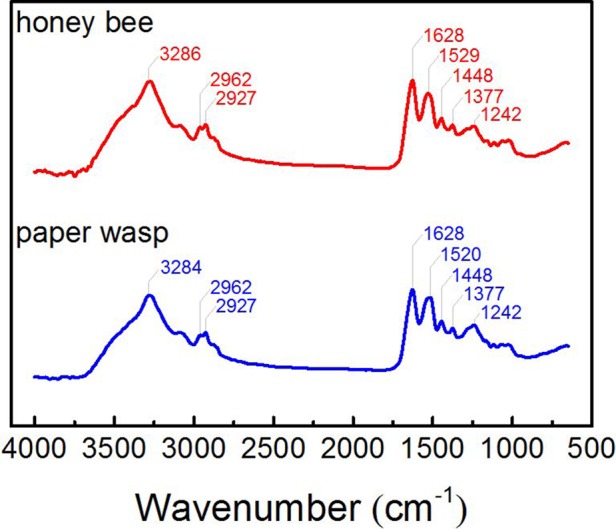
**Fourier transform infrared (FTIR) spectra of the stings of honey bees and paper wasps.** The chemical compositions of honey bee and paper wasp stings were determined from FTIR analysis with spectra in the optical range (wavenumbers) of 650–4000 cm^−1^ recorded at a resolution of 4 cm^−1^.

The FTIR spectra in Fig. 2 shows that the stings of honey bees and paper wasps basically have similar constituents, i.e. chitosan (Darmon and Rudall, 1950; Khan et al., 2002; Kumirska et al., 2010). The peak near 3284–3286 cm^−1^ corresponds to the –OH stretching vibrations, and the two peaks at 2962 cm^−1^ and 2927 cm^−1^ are attributed to the C–H stretching vibrations. The strong amide-I band at 1628 cm^−1^ indicates that the samples are the deacetylated derivative of *β*-chitin (Rinaudo, 2006). The absorption ratio of the intensity of amide-II band in the range of 1520–1529 cm^−1^ to that of the C–H stretching vibrations is commonly used to determine the degree of chitosan deacetylation (Kasaai, 2008). Besides, the peak at 1448 cm^−1^ is due to the bending of =CH_2_ and deformation of –CH_3_, and the peak at 1377 cm^−1^ is assigned to the bending of ≡CH and deformation of –CH_3_.

### Sting structures

**Fig. 3.**
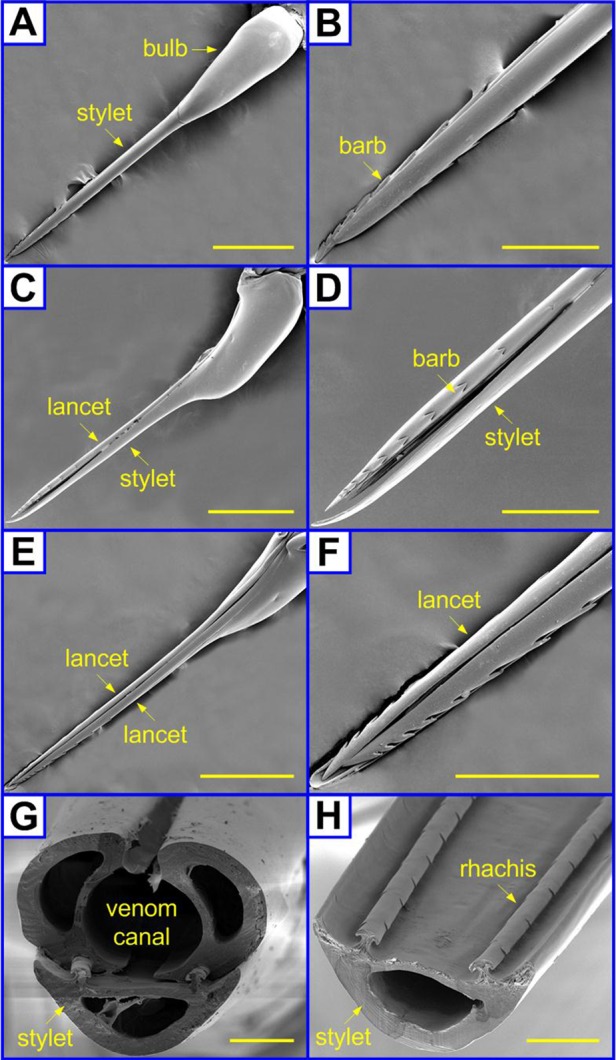
**Scanning electron microscopy of honey bee stings.** The microstructures of honey bee stings were observed using a scanning electron microscope. (A,B) Top, (C,D) lateral, (E,F) bottom views and (G,H) cross sections of the shaft of a honey bee sting. Scale bars=500 μm (A,C,E); 200 μm (B,D,F); 20 μm (G,H).

Fig. 3 shows the structures of a honey bee sting, which is comprised of three main components, including one stylet and two lancets. The honey bee sting is straight (Fig. 3A,C,E). Its stylet has a tapered tip, a bulb-like base, and a slender middle part with a nearly constant diameter of ∼86 μm (Fig. 3A). Fig. 3B is a magnified view of the stylet tip. The barbs on the stylet dorsal are substantially smaller than those of the lancets, which are shown in the supplementary material Fig. S1. The sub-apical barbs of the lancets are laterally protruded beyond the stylet. As can be seen from Fig. 3C, the lancet of a honey bee is barbed near its tapered tip. The sizes and spacings of the barbs on the lancets increase with their distance from the sting tip. The magnified SEM image in Fig. 3D reveals that all barbs on a lancet are located neatly along a straight line. Fig. 3E and F show that the two abreast lancets are arranged in tandem. To further observe its cross section, we cut the sting with a sharp blade. Fig. 3G shows that the stylet and lancets all have a hollow structure and a meniscus-like shape, surrounding a circular canal. Venom is usually stored in the basal bulb of a sting and it can be transported through the hollow canal. When the two lancets have a relative sliding, a gap appears at their tips (Fig. 3F), through which the venom can be pumped into the wounds (Dade, 1962). The stylet carries two rail-like protuberances (i.e. rhachises), which match well with the grooves (i.e. aulax) on the two lancets. A sliding interlocking mechanism is found in the rail-groove structure consisting of the stylet and lancets. By removing the lancets from the shaft, the rails on the stylet can be clearly revealed. Fig. 3H shows the existence of equidistant notches on the rails.

**Fig. 4.**
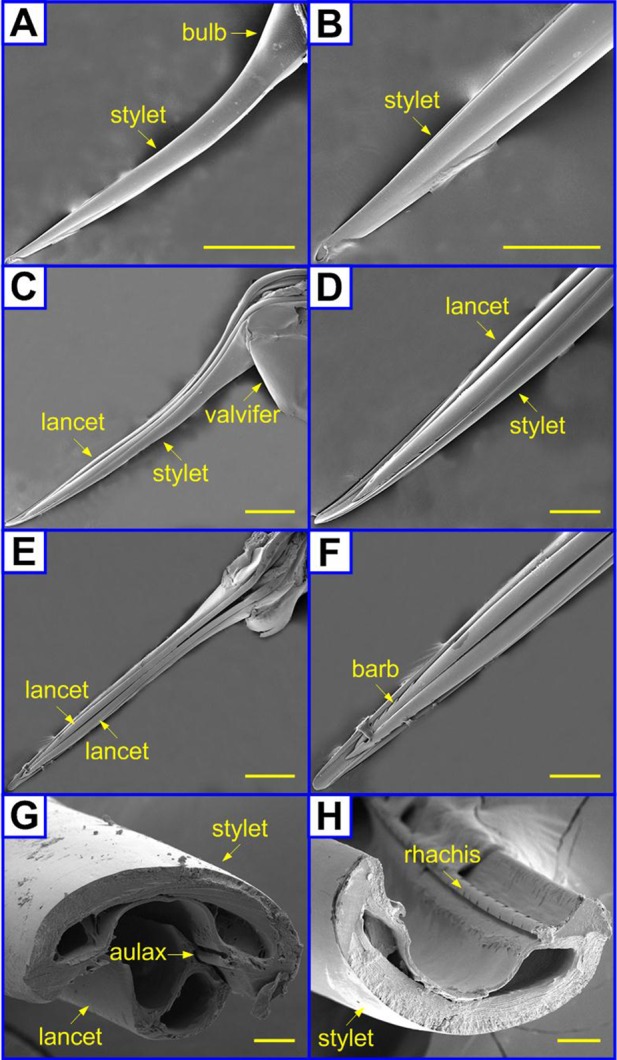
**Scanning electron microscopy of paper wasp stings.** The microstructures of paper wasp stings were observed using a scanning electron microscope. (A,B) Top, (C,D) lateral, (E,F) bottom views and (G,H) cross sections of the shaft of a paper wasp sting. Scale bars=500 μm (A,C,E); 200 μm (B,D,F); 20 μm (G,H).

Fig. 4 shows the delicate structure of a paper wasp sting. It has a larger curvature than the honey bee stings. Fig. 4A and B give the SEM images of the dorsal surface of the stylet and its magnified tip part, respectively. Different from the honey bee stings, no barb of the lancets is observed in the top views of the sting, and the basal bulb of the stylet has no significant protuberance (Fig. 4C). The lancets of a paper wasp sting also have small and laterally stretched barbs (Fig. 4D). However, the barbs are not laterally protruded beyond the stylet. In contrast to the abreast pair of a honey bee sting, the lancets of the paper wasp sting are featured by a chirality (Fig. 4E,F). One lancet overlaps the other after they converge (supplementary material Fig. S2), which is similar to the stings of some other wasp species, e.g. yellowjackets (Akre et al., 1981). The spiral morphology of the lancets reduces their transverse span and helps to hide the barbs in the stylet ventral such that they are not hooked by tissue fibers. The cross sections of a sting and an individual stylet are given in Fig. 4G and H, respectively. Similar to a honey bee sting, the stylet of a paper wasp sting interlocks with the two lancets along its entire length. However, the stylet of a paper wasp appears flatter than that of a honey bee (Fig. 3H).

### Penetration–extraction tests

**Fig. 5.**
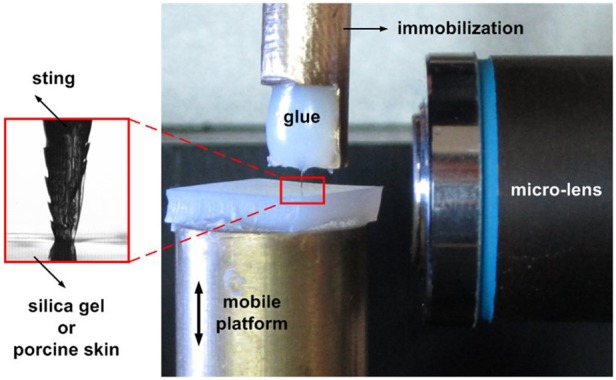
**Setup used in the penetration–extraction experiments.**

**Fig. 6.**
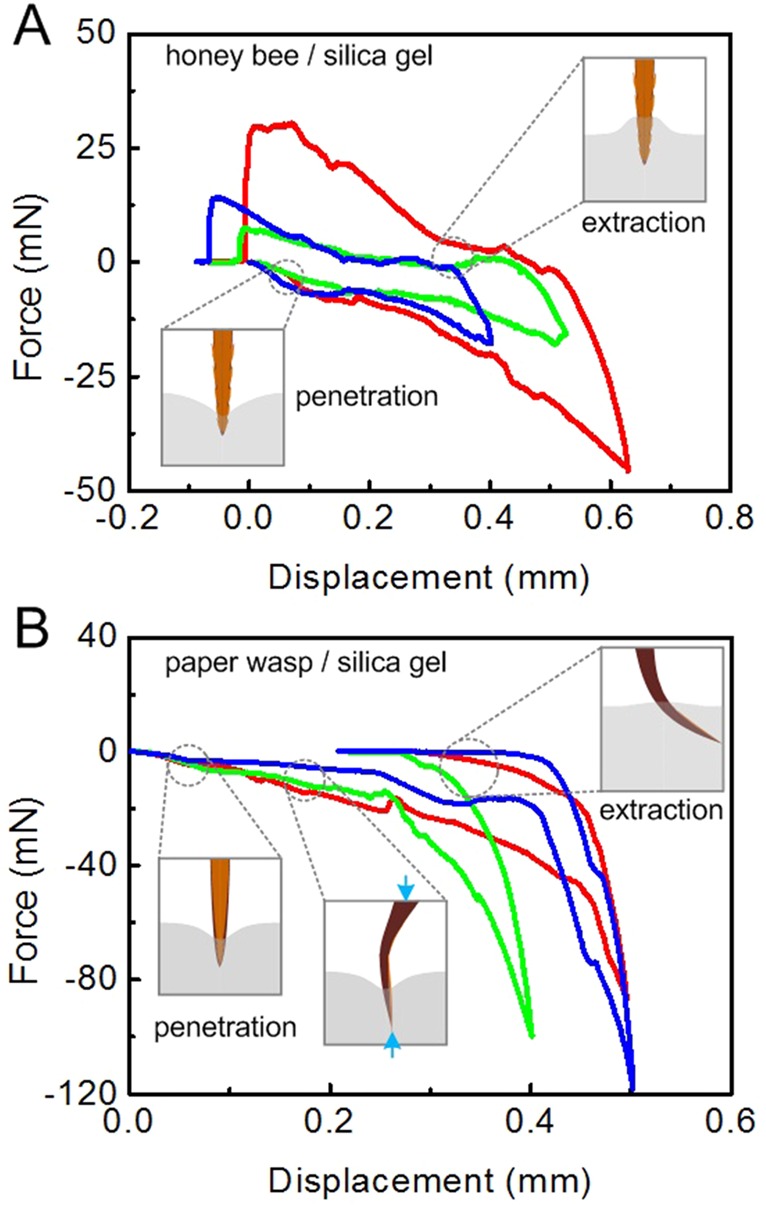
**Force–displacement curves from silica gel.** The force–displacement relation during the penetration–extraction process of the stings of (A) honey bees and (B) paper wasps inserting and pulling out from a silica gel was measured using a microforce test system with real-time motion of the sting synchronously recorded by using a CCD camera assembled with micro-lens.

To explore the relation between the penetration forces and the morphologies of the two kinds of stings, we performed penetration–extraction tests. The microforce test system used in our experiments is shown in Fig. 5. For the stings of honey bees and paper wasps piercing into the silica gel samples, three representative force–displacement curves measured during the whole penetration–extraction process are given in Fig. 6A and B, respectively. The inset figures schematize the typical configurations in the piercing-pulling out process. The silica gel starts to deform once the sting tip gets in contact with its upper surface. Then the penetration force increases steadily with the indentation depth. The work done by the sting is fully stored as the elastic strain energy in the substrate. The sting will pierce into the silica gel surface when the penetration force reaches a critical value. The force required to pierce into the substrate is referred to as the puncture force. The puncture forces are approximately 7–8 mN for honey bees (Fig. 6A) and 10–18 mN for paper wasps (Fig. 6B) for the stings inserting the silica gel, respectively. With a further increase in the applied force, the sting continuously cuts the substrate and the insertion depth increases. In this quasi-static piercing stage, the sting needs to deform and slice through the substrate and to overcome the incremental friction force. Thus, the penetration force continuously increases with depth. The force–displacement curves have some small fluctuations in both the penetration and retraction stages, which may be attributed to, e.g. the anchored sting barbs and the heterogeneity of the substrate. We denote the absolute values of the maximal penetration force and the maximal extraction force as *F*_p_ and *F*_e_, respectively. We here use the extraction–penetration force ratio *F*_e_/*F*_p_ to quantify the difficulty for a sting extracting from the substrate compared with that penetrating. The larger the force ratio, the more difficult the retraction. As shown in Fig. 6A, the force ratio of the honey bee sting interacting with silica gel is 0.61±0.19.

In comparison with the honey bee sting, the paper wasp sting was seriously bent during penetration. Due to its intrinsic curvature, the reaction force of the substrate acting on the sting is not aligned with the externally applied force at the basal part, as represented by the two blue arrows in the inset figures (Fig. 6B). Due to the bending moment induced by the two forces, the sting was apt to lodge in our tests, as shown in Fig. 6B. It is noticed that the straight penetration process in our tests is different from the real penetration manner of paper wasps, which pierce the substrate along a curved path, as we will show below.

**Fig. 7.**
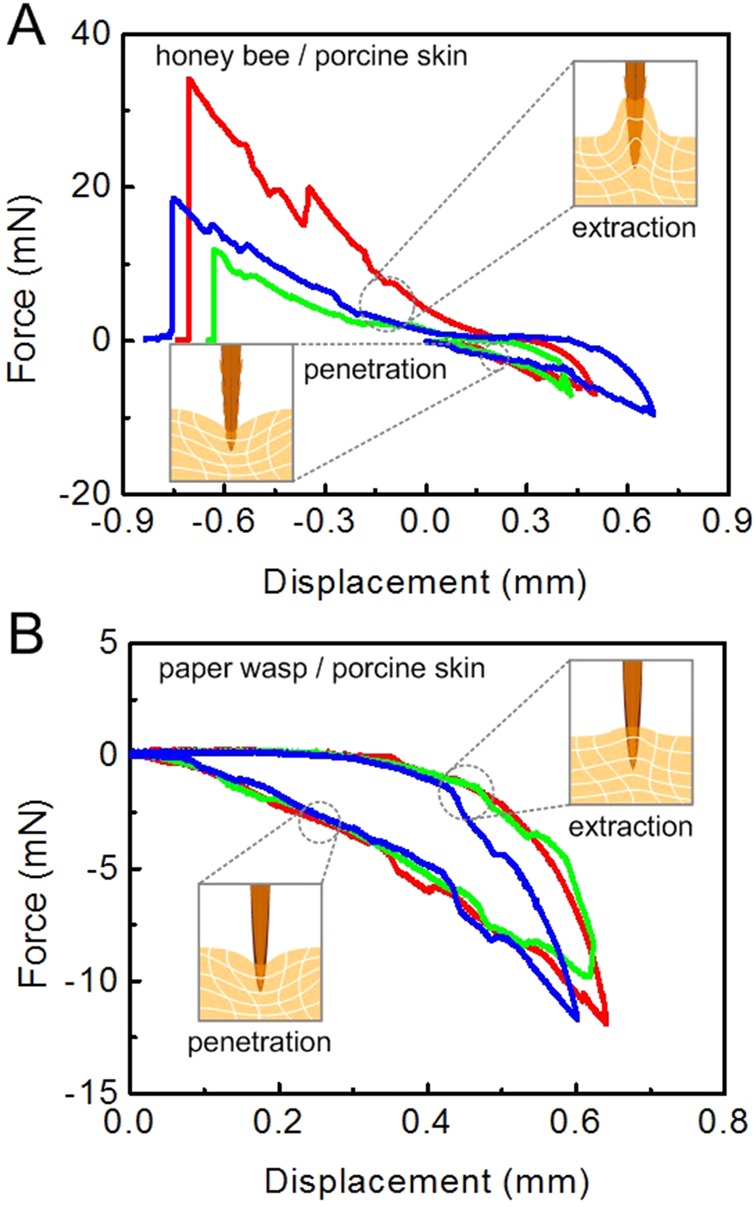
**Force–displacement curves from porcine skin.** The force–displacement relation during the penetration–extraction process of the stings of (A) honey bees and (B) paper wasps inserting and pulling out from porcine skin was measured using a microforce test system with real-time motion of the sting synchronously recorded by using a CCD camera assembled with micro-lens.

**Fig. 8.**
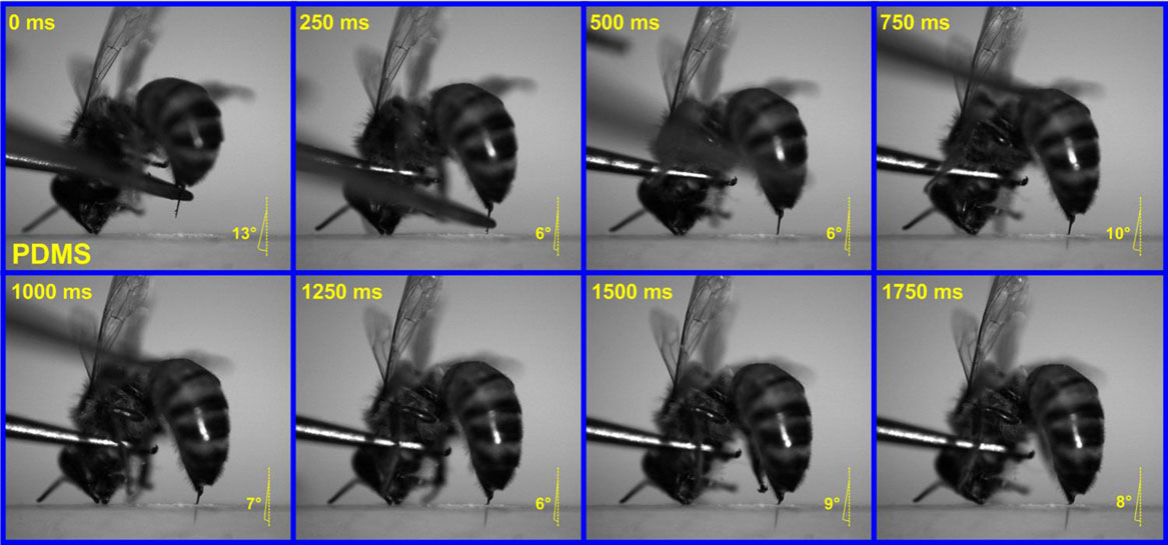
**Time-series graphs of a honey bee inserting PDMS bulk using its sting.** Slow motion analysis of a honey bee piercing a PDMS bulk was performed by using a high-speed video camera. The angles given indicate the penetration angle (*θ*) of the sting.

The penetration–extraction tests of stings were subsequently performed on a porcine skin, which was much softer than the silica gel and therefore easier to be penetrated. For the stings of honey bees and paper wasps inserting the porcine skin, the puncture forces were approximately 2–3 mN (Fig. 7A) and 6–8 mN (Fig. 7B), respectively. Unlike the isotropic and homogeneous silica gel, the dermis of the porcine skin contains collagen and elastic fibers and has a multilayer structure. The tissue fibers may interlock under the sting barbs, represented by the white curves in the insets of Fig. 7. If the barbs were anchored by tissue fibers, a larger force would be required to remove the sting from the porcine skin. This mechanism differs from the non-fibrous silica gel (Fig. 6). The forced extraction of the sting may not only bend its barbs but also damage the skin tissues. As shown in Fig. 7A, the force ratio *F*_e_/*F*_p_ for a honey bee sting inserting the porcine skin is 3.28±1.62, much higher than that inserting the silica gel. Therefore, it is more difficult to remove a honey bee sting from fibrous tissues (e.g. skin) than from non-fibrous substrates (e.g. silica gel). As the porcine skin is much softer, the paper wasp sting, albeit with a large curvature, can pierce into the porcine skin without buckling. Fig. 7B shows that the force ratio *F*_e_/*F*_p_ of the paper wasp stings is very small, suggesting its easy removal from the substrate. This indicates that their barbs were not anchored by tissue fibers.

### Slow motion analysis of insertion skills

The insertion skills of honey bee stings and paper wasp stings were further investigated by slow motion analysis. The penetration behavior of a honey bee sting was recorded as time-series graphs, as shown in Fig. 8 and supplementary material Movie S4. The honey bee had entirely thrust out its sting before it got in contact with the PDMS substrate. As can be seen from the first graph (0 ms), a drop of venom had already been extruded at the sting tip. The straight sting of the honey bee is approximately perpendicular to the surface of the victim during insertion. The whole piercing process lasted ∼1.5 s. Its penetration angle, *θ*, was labeled in each of the time-series graphs. During the piercing process, *θ* varies in a small range of a few degrees. At the end of insertion, the sting shaft had entered the substrate while its basal bulb remained out of the wound.

**Fig. 9.**
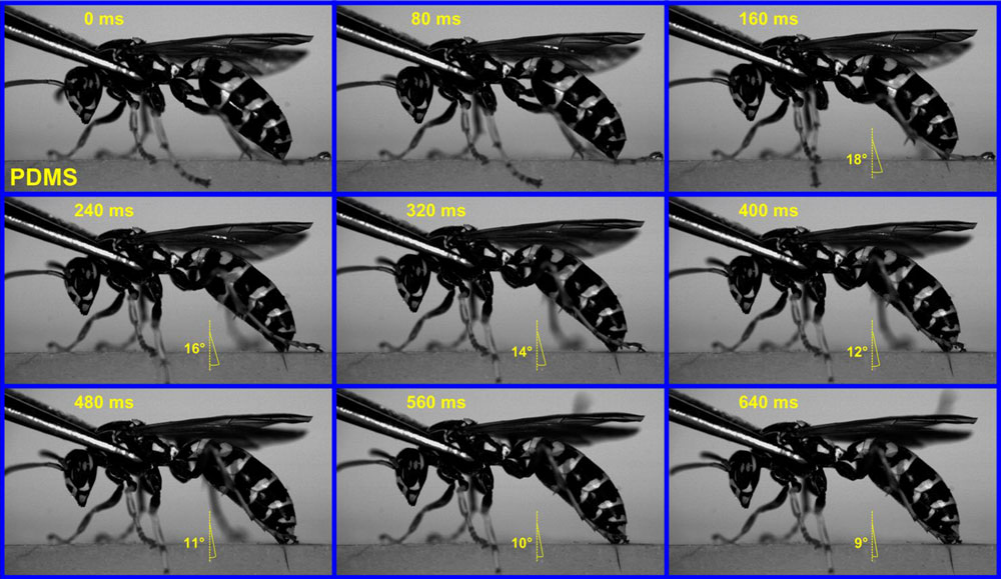
**Time-series graphs of a paper wasp inserting PDMS bulk using its sting.** Slow motion analysis of a paper wasp piercing a PDMS bulk was performed by using a high-speed video camera. The angles given indicate the penetration angle (*θ*) of the sting.

The time-series graphs of the penetration of a paper wasp sting into the PDMS bulk are shown in Fig. 9 and supplementary material Movie S5. The paper wasp firmly gripped the edge of the substrate with its barbed legs. Its sting was thrust out by elevating the oblong and quadrate plates. At the beginning of insertion, the sting tip was skewed to the substrate surface. During the piercing, the penetration angle *θ* continuously decreased from approximately 18° to 9°. The sting of the paper wasp had been adjusted to be approximately normal to the surface of the wounds at the end of penetration. It is also found that the originally curved sting was straightened when submerged into the PDMS bulk. It took only ∼0.5 s for the paper wasp to complete the penetration, which is ∼1/3 of the time spent by the honey bee.

## DISCUSSION

Both honey bees and paper wasps are members of the order Hymenoptera and suborder Apocrita. Being well-known pollinators, honey bees feed on pollen and nectar and attack when provoked or threatened. While paper wasps, preying upon or parasitizing other insects and scavengers (e.g. caterpillars, flies, and beetle larvae), and sometimes sipping on nectar, are more aggressive predators. Therefore, a paper wasp uses its sting more frequently than a honey bee. Our experiments reveal that the stings of honey bees and paper wasps, though with similar biological functions and chemical constituents, have evolved distinctly different structures and mechanical behaviors. Their stings, derived from ovipositors, have multiple functions, e.g. attack, defense, and prey carriage. FTIR spectra indicate that both the stings of honey bees and paper wasps consist mainly of chitosan. As a deacetylated derivative of chitin, chitosan widely exists in, e.g. bacteria, animals, and plants (Rinaudo, 2006). It is a key constituent in the exoskeleton of diverse crustaceans, e.g. crayfishes, shrimps and crabs (Zhao et al., 1998), and contributes to their superior mechanical properties, e.g. high elastic modulus and toughness. It is noted that the stings of honey bees and paper wasps are both flexible because of their small diameters and hollow structures. It is difficult for us to pierce them into, for example, a porcine skin, after the insects have been dead. The sting shaft possesses an elegant microstructure and the insects themselves have mastered refined insertion skills, for example, regulation of the piercing direction. Each sting has three main components, including one stylet and two lancets. Resorting to the interlocking mechanism, the lancets can slide freely on the two rails of the stylet. The stylet with a bevel tip can easily wedge the wounds. The lancets have hollow structures, rending efficient material utilization and improved mechanical properties.

The stings of both honey bees and paper wasps are featured by barbs. The lancets literally saw through the victim's fresh as each in turn is thrust forward and anchored in place by their barbs (Akre et al., 1981). The barbs have unique biological and mechanical functions, for example, to help efficiently hold preys and prevent them from slipping off the sting. During the insertion of a sting into a tissue, the barbs may reduce the penetration force through two main mechanisms. First, owing to the barb-induced stress concentration in the tissue, the sting can cut the tissue more easily (Cho et al., 2012). Second, tissue fluids can be squeezed out at the barb positions and serve as lubricants. Thereby, the decreased coefficient of friction helps to reduce the penetration force.

The Janus-faced barbs of a honey bee sting, albeit their significance to some advanced biomechanical functions, may result in a difficult removal. During the penetration of a honey bee sting into a fibrous tissue (e.g. skin), its barbed lancets saw their way into the wound. The barbs may interact with the substrate and be anchored by tissue fibers, making the sting difficult to be withdrawn. When the honey bee needs to pull away, its sting may be lodged and torn loose from the abdomen, or even ripped out together with some internal organs and left in the victim tissue, if the tissue has higher elastic modulus and strength. The honey bee will die within a few hours or days (Haydak, 1951) due to the massive abdominal rupture. Our penetration–extraction tests on silica gel demonstrate that the barbs also lead to a difficult removal of the sting from a non-fibrous substrate. By contrast, the stings of paper wasps can be repeatedly used to penetrate both non-fibrous and fibrous tissues.

During its insertion into a victim, the honey bee adjusts its posture by bending and twisting the abdomen. The sting has been entirely thrust out before getting in contact with the victim surface and is like a nail ready for driving in. Due to its large slenderness, the sting is vulnerable to axial buckling. Therefore, the honey bee continuously tunes the penetration angle in order to prevent the sting from buckling. The barbs on the lancets also play an important role in the penetration process of the sting. The row of barbs is skewed to the sting axis with an angle of ∼8°, and correspondingly the sting has an axial rotation during penetration (Wu et al., 2014). The helical rotation helps the sting tip bypass the tissue fibers or hard components, rendering an easier piercing. This refined penetration skill of honey bees is somewhat like the acupuncture and moxibustion in traditional Chinese medicine therapy.

Different from the straight morphology of honey bee stings, paper wasp stings have a relatively large intrinsic curvature. A paper wasp would not thrust its sting out until it gets in contact with the victim surface. Differing from the honey bee sting, which pierces into the substrate like a straight nail, a paper wasp sting penetrates a materials along a curved or arc path. A paper wasp sting has a reinforcing rib in the middle of the stylet ventral, which improves its buckling resistance (supplementary material Fig. S3). While the stylet of a honey bee sting does not have such a reinforcement (supplementary material Fig. S4). At the beginning of insertion, the paper wasp adjusts its sting forepart to skew into the victim surface. In the penetration–extraction tests, the sting forepart is mounted to keep perpendicular to the substrate surface and the sting base is clamped and rotation prohibited. Due to the intrinsic curvature, the basal part and forepart of the sting have an inclined angle with respect to the substrate surface. Therefore the curved sting is subjected to a bending moment and may axially lodge during its insertion of the relatively stiff silica gel. In order to prevent the sting from buckling, the paper wasp gradually decreases its penetration angle during insertion. The barbs on the paper wasp stings are much smaller than those on the honey bee stings. To avoid their barbs being anchored by tissue fibers, the lancets assume a spiral shape. When submerged in the victim, the originally curved sting will be straightened. The barbs, hidden in the broad stylet, have little interaction with the substrate. In comparison with the honey bee stings, the paper wasp stings are easier to be extracted from the wound. Therefore, both the intrinsic curved shape of the sting and the spiral morphologies of its two lancets are crucial for the paper wasp stings to rapidly penetrate into and readily extract from the attacked body.

Following the above results and discussions, some similarities and differences of the stings of honey bees and paper wasps are summarized in Table 1.

**Table 1.**
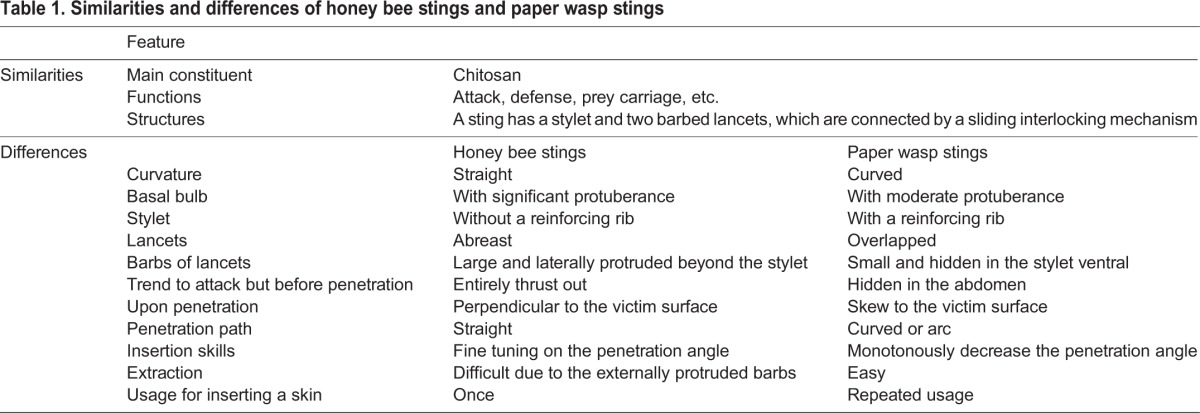
**Similarities and differences of honey bee stings and paper wasp stings**

## CONCLUSION

We have experimentally investigated and compared the stings of honey bees and paper wasps from the viewpoints of chemical compositions, geometric morphologies, and biological functions. Both kinds of stings are mainly comprised of chitosan, and each sting has a stylet and two barbed lancets, which are connected by a sliding interlocking mechanism. The honey bee stings are relatively straight and have laterally stretched barbs, while the paper wasp stings have an intrinsic curved shape and have smaller barbs hidden in the stylet. The paper wasp stings can be easily retracted from the victim substrate, while the removal of the honey bee stings is more difficult due to their externally protruded barbs. Both honey bees and paper wasps have refined insertion skills adaptive to their stings with different sizes and morphologies. This study might be helpful to gain insights into the relations among the chemical compositions, geometric structures, mechanical properties, and biological functions of the insect stings. The results may also provide inspirations for the design of bioinspired stings and microneedles.

## MATERIALS AND METHODS

The care and use of the experimental animals complied with the institutional and national animal welfare laws, guidelines, and policies. Honey bees (*Apis cerana* Fabricius) and paper wasps (*Polistes sp.*) were collected at Beijing. Fig. 1 shows a honey bee, a paper wasp, and the stings of the two species at the maximum thrust, which are indicated by the yellow arrows. The masses and body lengths of 10 honey bees and 10 paper wasps randomly selected were measured. The mass was determined by an electronic balance, and the body length was measured from the forehead to the tip of abdomen by using a digital caliper.

The stings of honey bees and paper wasps were taken out from the fresh cadavers by extruding their abdomens with a pair of forceps. The stings were submerged in distilled water, cleaned by the ultrasonic method, and then naturally dried in airtight glassware. To determine their chemical compositions, the Fourier transform infrared (FTIR) spectra of the stings were obtained by using a Nicolet 6700 FTIR spectrophotometer (Nicolet Instrument Company, USA). The spectra in the optical range (wavenumbers) of 650–4000 cm^−1^ were recorded at a resolution of 4 cm^−1^. The sting microstructures were observed using scanning electron microscope (SEM, Quanta FEG 450, FEI, USA). The stings were cut by sharp blades for cross-section observations. The different components in each sting were separated from each other with a forceps in order to observe their more detailed structures. All samples were gold sputtered before observation.

The quasi-static penetration–extraction tests of the stings were carried out on a silica gel (non-fibrous substrate) and a piece of porcine skin (fibrous tissue) in the dorsal region. The silica gel samples, with Young's modulus 1–2.8 MPa and tensile strength 3.5–15 MPa (Shergold and Fleck, 2005), were made into a cuboid of 2 mm in depth. The fresh skin samples, obtained by removing the subcutaneous tissues, were roughly comprised of epidermis and dermis. The Young's modulus and tensile strength of the skin samples are 0.3–1.0 MPa and 10–20 MPa, respectively (Shergold and Fleck, 2005). A microforce test system (JQN04C, Powereach, China) was used to examine the force–displacement relation during the penetration–extraction process of a sting. The sting was immobilized at the upper end, while the silica gel/porcine skin was fixed on the mobile platform. Insertion and pull-out of the sting were controlled by moving the platform upwards and downwards, respectively. The real-time motion of the sting was synchronously recorded by using a CCD camera assembled with micro-lens. Before the tests, the sting foreparts were adjusted to be perpendicular to the substrate surface through the micro-lens. The loading rate was set as 1 μm s^−1^.

The slow motion analysis of the penetration behaviors of honey bees and paper wasps was performed by using a high-speed video camera (Fastcam Mini UX100, Photron, Japan). The honey bee and paper wasp, handled by a forceps, were guided to pierce a polydimethylsiloxane (PDMS) bulk by, e.g. alarm pheromone and physical stimulation. The PDMS substrate was prepared by mixing the curing agent (Sylgard 184, Dow Corning, USA) and base with a weight ratio of 1:30. The mixture was degassed for 1–2 h to remove excess bubbles, and cured at 60°C for 3 h. The Young's modulus of the PDMS (1:30) bulk is 0.3±0.01 MPa (Lim and Chaudhri, 2004). The PDMS (1:30) bulk was semi-transparent such that the sting motion could be directly observed and recorded. The insertion skills of honey bees and paper wasps were examined by comparing their features of penetration. The angle of the sting deviated from the normal of the substrate surface, referred to as the penetration angle *θ*, was measured.

## Supplementary Material

Supplementary Material
