# Supplementary material for: Structures, properties, and functions of the stings of honey bees and paper wasps: a comparative study
Source: Biol Open. 2015 May 22;4(7):921–8. doi: 10.1242/bio.012195 (PMC4571097; doi:10.1242/bio.012195)
Supplement: Supplementary Material [file supp_4_7_921__index.html]

Structures, properties, and functions of the stings of honey bees and paper wasps: a comparative study — Structures, properties, and functions of the stings of honey bees and paper wasps: a comparative study — Supplementary Material 

# Structures, properties, and functions of the stings of honey bees and paper wasps: a comparative study

## BIO012195 Supplementary Material

- Supplementary Material
