# Supplementary material for: Structures, properties, and functions of the stings of honey bees and paper wasps: a comparative study
Source: Biol Open. 2015 May 22;4(7):921–8. doi: 10.1242/bio.012195 (PMC4571097; doi:10.1242/bio.012195)
Supplement: Supplementary Material [file supp_bio.012195_BIO012195supp.pdf]

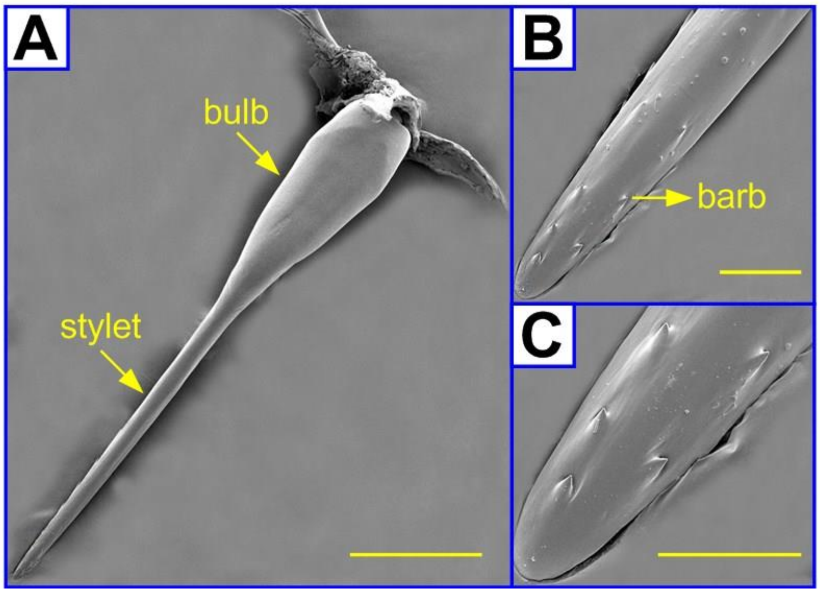

Fig. S1: (A) Stylet dorsal of a honey bee sting, and (B, C) magnified views of its distal barbs. The barbs on the stylet dorsal are distinctly smaller than those on the lancets. The scale bars are 500 μm for (A), 50 μm for (B), and 50 μm for (C).

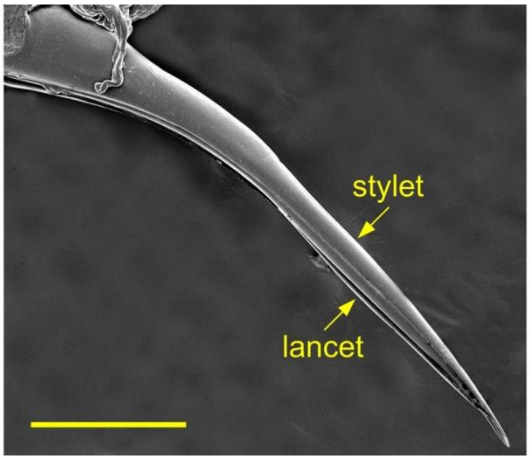

Fig, S2: Lateral view of a paper wasp sting. The sting has a distinct curvature. One lancet overlaps the other during their convergence. The scale bar is 500 μm.

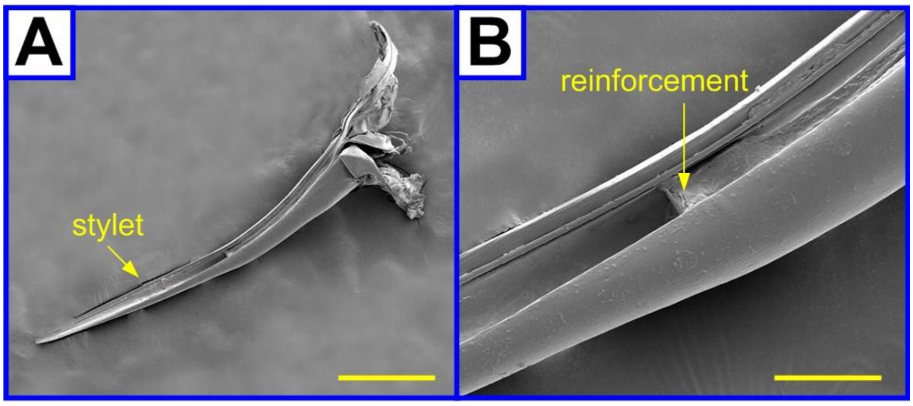

Fig. S3: (A) Stylet ventral of a paper wasp sting and (B) a magnified view of its middle part. The paper wasp sting has a reinforcing rib in the middle of the stylet ventral, which improves its buckling resistance. The scale bars are 500 μm for (A) and 100 μm for (B).

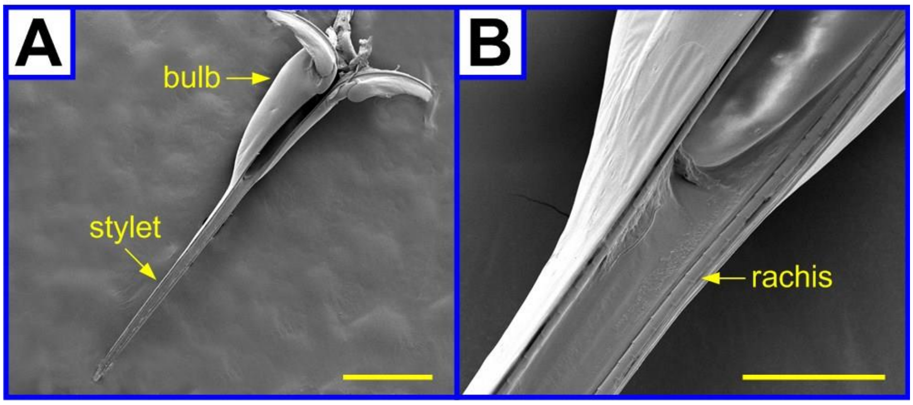

Fig. S4: (A) Stylet ventral of a honey bee sting and (B) a magnified view of its middle part. The sting has no reinforcement at the middle of the stylet. The scale bars are 500 μm for (A) and 100 μm for (B).

Movies

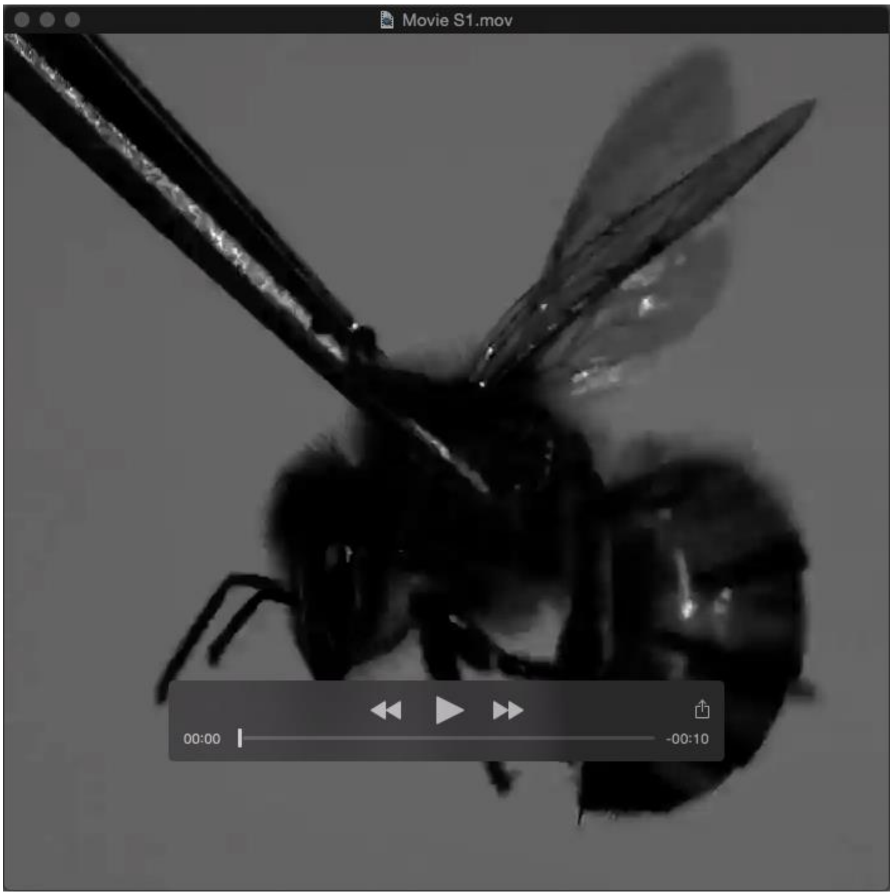

Movie S1: Slow motion of a honey bee thrusting out its sting.

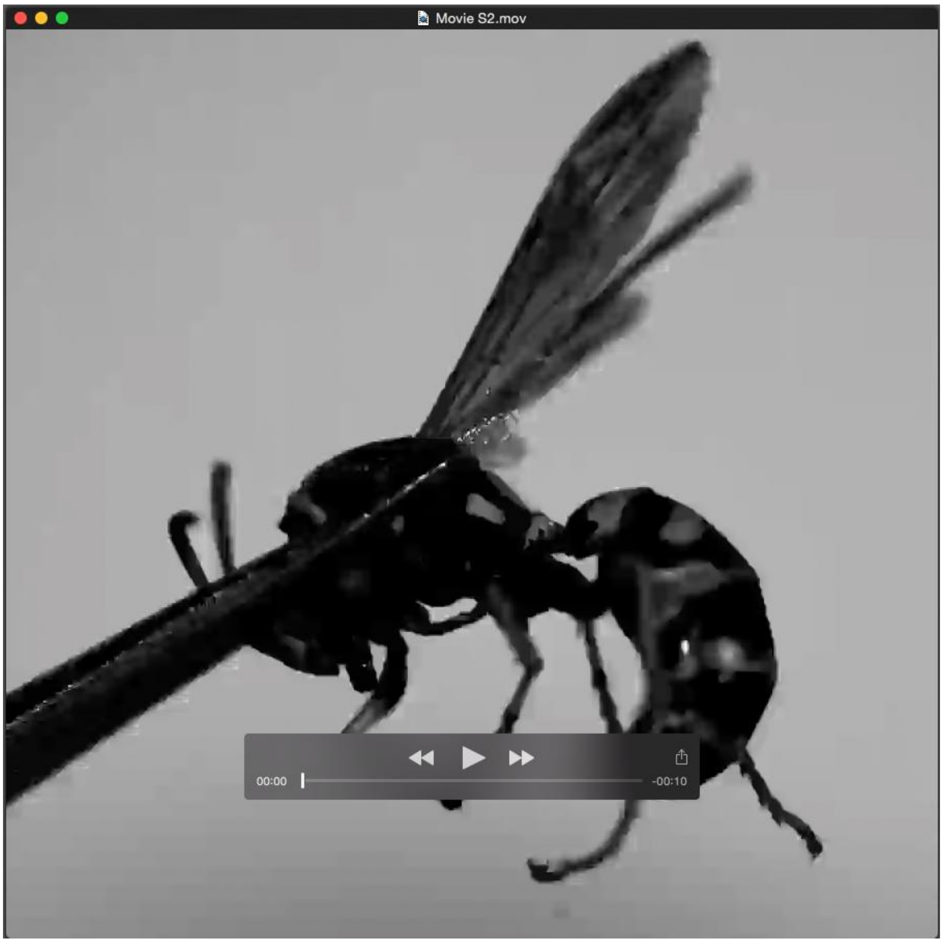

Movie S2: Slow motion of a paper wasp thrusting out its sting.

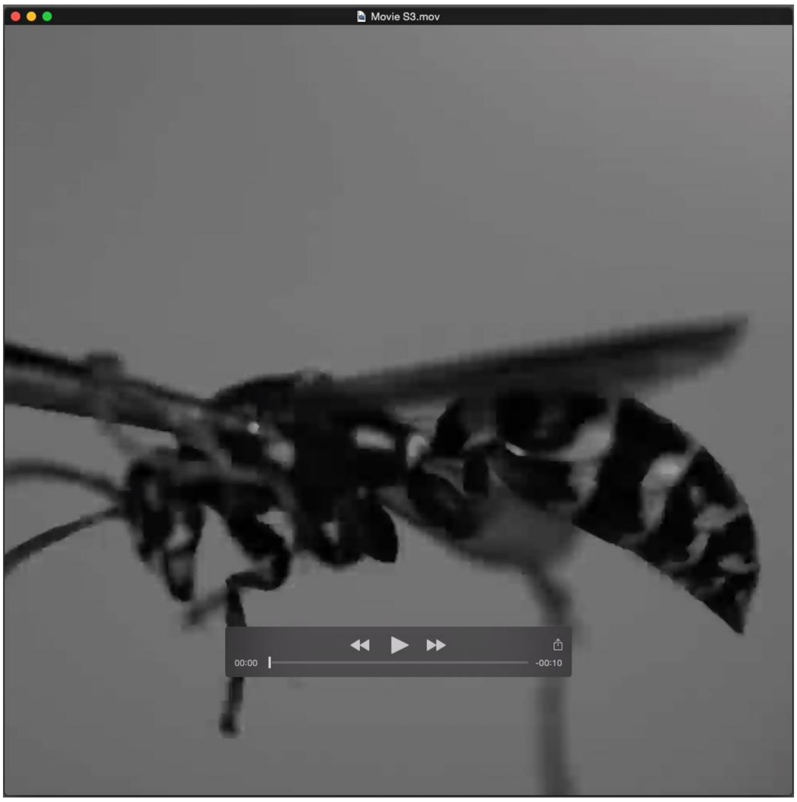

Movie S3: Slow motion of a paper wasp spinning and bending its abdomen and thrusting out its sting.

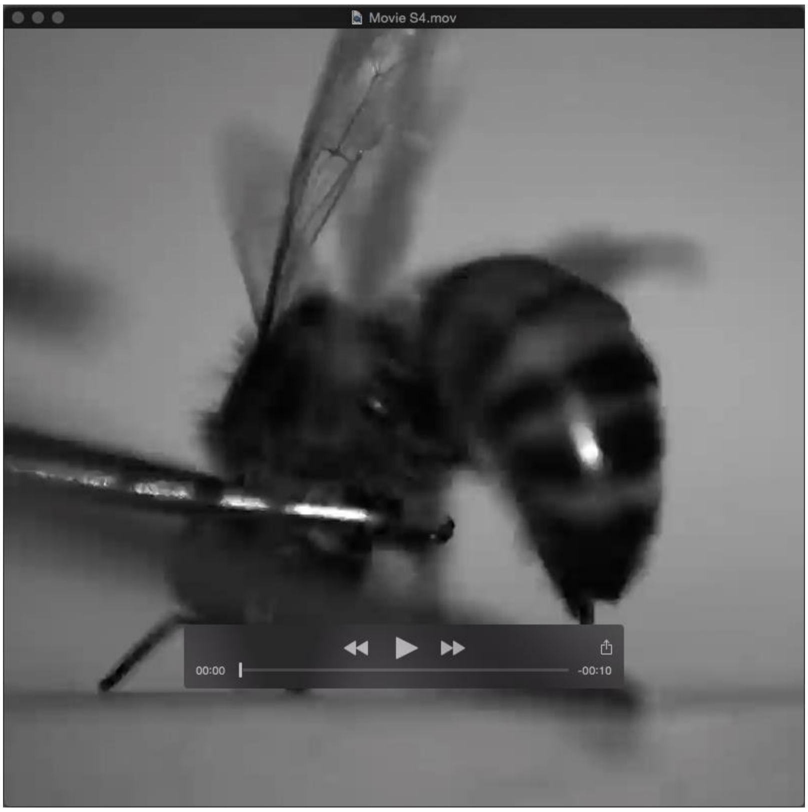

Movie S4: Slow motion of a honey bee inserting PDMS bulk using its sting.

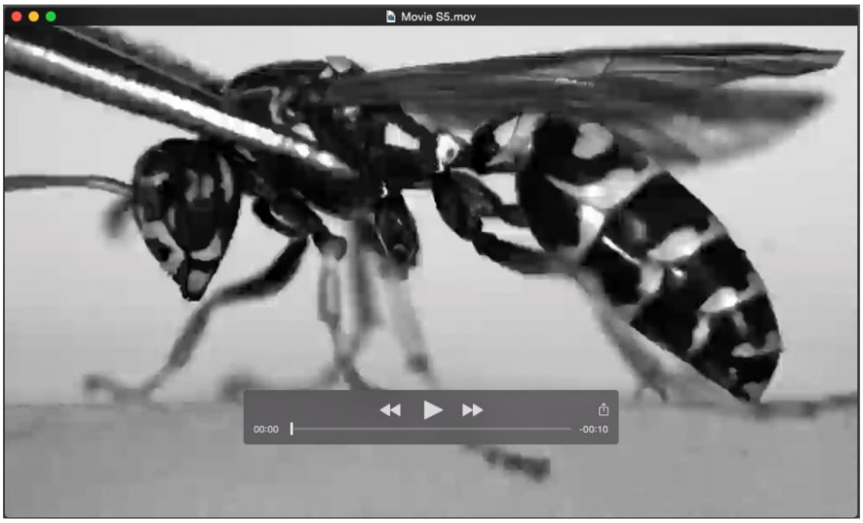

Movie S5: Slow motion of a paper wasp inserting PDMS bulk using its sting.
